# Tailoring vertical phase distribution of quasi-two-dimensional perovskite films via surface modification of hole-transporting layer

**DOI:** 10.1038/s41467-019-08843-5

**Published:** 2019-02-20

**Authors:** Tiefeng Liu, Youyu Jiang, Minchao Qin, Junxue Liu, Lulu Sun, Fei Qin, Lin Hu, Sixing Xiong, Xueshi Jiang, Fangyuan Jiang, Ping Peng, Shengye Jin, Xinhui Lu, Yinhua Zhou

**Affiliations:** 1 Wuhan National Laboratory for Optoelectronics, and School of Optical and Electronic Information, Huazhong University of Science and Technology, Wuhan, 430074 China; 2 Department of Physics, The Chinese University of Hong Kong, New Territories, Hong Kong, 999077 China; 3 State Key Laboratory of Molecular Reaction Dynamics and Collaborative Innovation Center of Chemistry for Energy Materials (iChEM), Dalian Institute of Chemical Physics, Chinese Academy of Sciences, 457 Zhongshan Road, Dalian, 116023 China; 4 School of Materials Science and Engineering, Huazhong University of Science and Technology, Wuhan, 430074 China

## Abstract

Vertical phase distribution plays an important role in the quasi-two-dimensional perovskite solar cells. So far, the driving force and how to tailor the vertical distribution of layer numbers have been not discussed. In this work, we report that the vertical distribution of layer numbers in the quasi-two-dimensional perovskite films deposited on a hole-transporting layer is different from that on glass substrate. The vertical distribution could be explained by the sedimentation equilibrium because of the colloidal feature of the perovskite precursors. Acid addition will change the precursors from colloid to solution that therefore changes the vertical distribution. A self-assembly layer is used to modify the acidic surface property of the hole-transporting layer that induces the appearance of desired vertical distribution for charge transport. The quasi-two-dimensional perovskite cells with the surface modification display a higher open-circuit voltage and a higher efficiency comparing to reference quasi-two-dimensional cells.

## Abstract (web summary)

Vertical phase distribution of quasi-two-dimensional perovskite plays vital roles in their optoelectronic properties. Here Liu et al. show that surface modification of the hole-transporting layer is an effective approach to control the vertical phase distribution and optimize the device efficiency.

## Introduction

Three-dimensional (3D) organic-inorganic hybrid perovskites have drawn enormous research interest in the past few years for photovoltaic applications due to their unique optoelectronic properties, such as high absorption coefficient^1^, small exciton binding energy^2^, long carrier diffusion lengths^3,4^, excellent charge mobility^5^, low defect densities^6,7^, and tunable bandgaps^8,9^. The highest power conversion efficiency (PCE) of 3D perovskite solar cells has reached 23.7%^10^. However, the chemical activity and instability of perovskite^11–14^, especially over moisture^15,16^, restrict its development and applications.

In recent years, the layered quasi-two-dimensional (Q-2D) (A_1_)_2_(A_2_)_*n−*1_B_*n*_X_3*n*+1_ [where A_1_ is large-size bulky organic cation, such as butylammonium (BA) or phenethylammonium (PEA); A_2_ is Cs, methylammonium (MA) or formamidinium (FA); B is Pb or Sn; X is halide anion; and the integer *n* is number of layered inorganic [PbI_6_] sheets between two bulky organic cation spacers] perovskites have shown potential to improve the physico-chemical stability^17–20^. The hydrophobic feature of longer alkyl chain in bulky organic cation^21–23^ and the strong van der Waals interaction between the capping organic molecules and the [PbI_6_] unit^24^ contributes to the enhancement of stability. The large-sized organic cations in the Q-2D structure isolate the electronic coupling between the *n*-layer [PbI_6_] sheets, resulting in the formation of the multi-quantum-wells in the Q-2D films. Photo-generated excitons are confined within the layered crystalline lattice in the multi-quantum-wells which leads to larger exciton binding energy than that of their 3D counterparts^25,26^. The insulating organic spacers will also inhibit the out-of-plane charge transport^27,28^. To ensure the efficient collection of photo-generated charge carriers, hot-casting deposition approach^29^, solvent engineering^30–32^, additive engineering (SCN^*−*^, MACl)-^33–35^, and composition optimization (FA, Cs)^36,37^ have been adopted to facilitate the vertical growth. Currently, the PCE for small-*n* Q-2D (*n* = 4) perovskite solar cells has reached 12.81% (inverted structure)^36^ and 13.68% (conventional structure)^37^. It is still lower than that of 3D perovskite solar cells.

Different from their 3D counterparts, previous reports have shown that the fabricated Q-2D perovskite films are actually a mixture of multiple perovskite phases with different *n* values instead of a homogeneous perovskite phase with an identical *n* value. These multiple phases are arranged spontaneously along the direction perpendicular to the substrate from small-*n* to large-*n* (from substrate side to the top surface of films)^38,39^. Jin and co-workers have demonstrated that such multiple-phase structure in Q-2D perovskite films contain desired energy band alignment, which benefits the electron-hole separation and facilitates the charge transfer between the phases with small-*n* and large-*n*. The vertical energy alignment from small-*n* to large-*n* is shown in the Supplementary Figure 1 (note: the energy levels of the Q-2D perovskite with different *n* are extracted from the work reported by Kanatzidis^21^ where the energy level measurements are performed on homologous 2D perovskite crystals). The internal charge transfer property facilitates the charge extraction to the electrodes for photovoltaic applications in inverted device structure^38,40,41^.

In this work, we find that the desired vertical phase distribution of *n* from the small to the large disappears when the Q-2D perovskite is deposited on the hole-transporting layer (HTL) of poly(3,4-ethylenedioxythiophene): poly(styrene sulfonate) (PEDOT:PSS), which is different from that on the glass substrate. We employ a self-assembly layer of 4-bromobenzenediazonium tetrafluoroborate covalently anchored onto the surface of the PEDOT:PSS that can induce the appearance of the desired vertical phase distribution of *n* in the Q-2D perovskite films. The PEDOT:PSS modified by 4-bromobenzenediazonium tetrafluoroborate is henceforth referred to as BrB-PEDOT:PSS. The devices with BrB-PEDOT:PSS display reduced non-radiative recombination and higher *V*_OC_ (1.11 V) comparing to the reference Q-2D perovskite cells without the surface anchoring layer. The (BA)_2_(MA_0.95_Cs_0.05_)_3_Pb_4_I_13_ Q-2D cells with the BrB-PEDOT:PSS HTL show a PCE of 13.74%.

## Results

### Observation of vertical phase distribution and tailoring

**Fig. 1:**
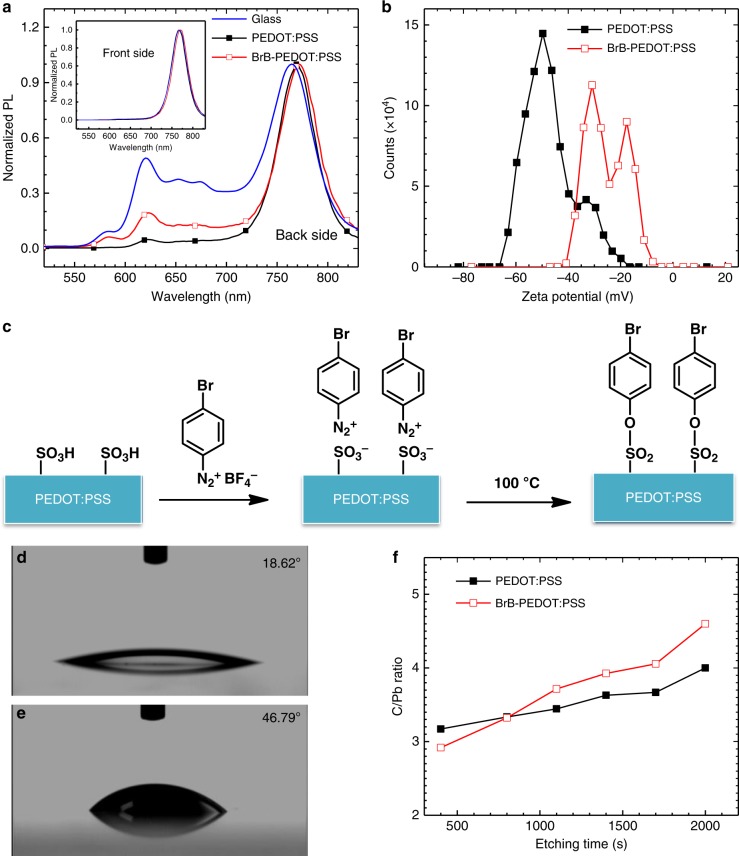
Observation of vertical phase distribution and tailoring in Q-2D perovskite films. **a** PL spectra of Q-2D perovskite films on different substrates excited from the back side (substrate side). The inset is the PL spectra of Q-2D perovskite films on different substrates excited from the front side; **b** the zeta potential of PEDOT:PSS and BrB-PEDOT:PSS; **c** the schematic of covalent modification on PEDOT:PSS via a molecule of 4-bromobenzenediazonium tetrafluoroborate. Contact angle images of water on: **d** PEDOT:PSS and **e** BrB-PEDOT:PSS. **f** XPS depth profiles of C/Pb atom ratios as a function of etching time for the Q-2D films. (etching direction: surface of the perovskite film to the substrate side)

We began with the study on the vertical phase distribution of Q-2D perovskite on glass substrate using photoluminescence (PL) spectroscopy. BA is used for the large-size cations to generate Q-2D perovskite layers. To enhance the films quality, 5% Cs was added into the Q-2D perovskite precursors^37^. The stoichiometry of precursor determines the chemical structure of Q-2D perovskite is (BA)_2_(MA_0.95_Cs_0.05_)_3_Pb_4_I_13_. PL measurements were excited from both the front side (perovskite surface) as well as the back side (glass side). As shown in Fig. 1a, when excited from the front side, the PL spectrum shows a single emission peak at about 770 nm (the insert of Fig. 1a). When excited from the back side, multiple emission peaks at higher energy appear. Such difference observed in the PL spectrum is consistent with the previous reports^38,39^. It suggests that there are small-*n* phases at the bottom of the perovskite films near the substrate and large-*n* phases at the upper surface of the perovskite film. This vertical phase distribution is favorable for charge separation and transport in inverted perovskite solar cell structure^35,38,39^.

Then, we tried to build this vertical phase distribution on PEDOT:PSS films that are typically used as HTL in the inverted Q-2D perovskite solar cells. However, it is surprising that the multiple emission peaks associated with small-*n* phases at the bottom side nearly disappear (Fig. 1a) when the Q-2D perovskite films are grown on PEDOT:PSS layer with the same film casting method. The PEDOT:PSS suppresses the vertical phase distribution of the Q-2D perovskite.

To obtain the desired vertical phase distribution on PEDOT:PSS HTL, we use a small molecule of 4-bromobenzenediazonium tetrafluoroborate to react with PEDOT:PSS (BrB-PEDOT:PSS) to tune the surface properties of the PEDOT:PSS. Through the interaction between the diazo cation and –SO_3_^−^ group followed by post-annealing, the ionic bond is converted to covalent bond (Fig. 1c). This covalent modification was verified using the Fourier transform infrared (FT-IR) spectrum (Supplementary Figure 2). The symmetric stretching band of O = S = O changes from 1176 (in PEDOT:PSS) to 1172 cm^−1^.(in BrB-PEDOT:PSS). That suggests there exists the conversion of –SO_3_^−^ (ionic bond) to –SO_2_–O– (covalent bond)^42,43^. After modification, the zeta potential of the PEDOT:PSS shifts from −53 to −29 mV (Fig. 1b) and the contact angle increases from 18.62° to 46.79° (Fig. 1d, e). The shift of the zeta potential also confirms the conversion of ionic bond to covalent bond where less negative ions exist after the reaction. The larger contact angle further confirms the modification. Importantly, the small-*n* peaks of Q-2D perovskite films deposited on BrB-PEDOT:PSS become more pronounced compared to that on PEDOT:PSS (Fig. 1a). The Q-2D perovskite films on both HTLs have similar absorption spectrum (Supplementary Figure 3).

To further investigate the compositional gradient BA and MA in the Q-2D perovskite films, we used depth-profiling X-ray photoelectron spectroscopy (XPS) to detect the C/Pb atom ratio where the number of C atom in BA is larger than that in MA. As etching time increases, the C/Pb ratio becomes larger when the substrate is BrB-PEDOT:PSS, as shown in Fig. 1f, which indicates that the Q-2D perovskite films fabricated on the BrB-PEDOT:PSS have more BA located at the bottom of perovskite film (close to the substrate side) compared to those fabricated on the PEDOT:PSS. That is consistent with the results of PL spectra.

### Understanding the origin of vertical phase distribution

**Fig. 2:**
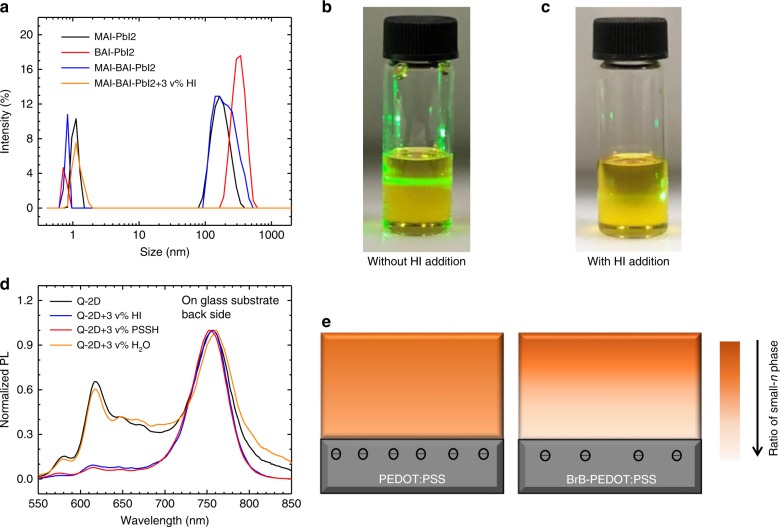
Origin of vertical phase distribution in the Q-2D perovskite films. **a** Colloid size distribution of perovskite precursors in GBL:DMSO mixed solvents. The mole ratio of MAI-PbI_2_ is 1:1, presenting the target *n* is infinity. The mole ratio of BAI-PbI_2_ is 2:1, presenting the target *n* is 1. The mole ratio of MAI-BAI-PbI_2_ is 3:2:4, presenting the target *n* is 4. MAI-BAI-PbI_2_ + 3 v% HI denotes 3 vol.% HI is added into the precursor of MAI-BAI-PbI_2_ (3:2:4). Tydall effect photographs of the Q-2D perovskite precursors: **b** without or **c** with 3 vol.% HI addition. **d** PL spectra of Q-2D perovskite films (with or without acid addition) deposited on glass substrates excited from back side. **e** Schematic of phase distribution (small-*n* to large-*n* phase from substrate side to the top side) in Q-2D perovskite films on PEDOT:PSS and BrB-PEDOT:PSS substrates

The vertical phase distribution with different *n* values is dependent on the distribution of large-size capping cation of BA in the Q-2D perovskite films. In previous studies, the perovskite precursors have been reported in the form of colloids^44,45^. Here, the Tyndall effect of Q-2D perovskite precursor is shown in Fig. 2b that confirms the colloidal feature of the precursor. The vertical distribution of the colloids can be analyzed with the equation of sedimentation equilibrium:^46^

1 $$\frac{{c_2}}{{c_1}} = {\mathrm{e}}^{ - \frac{{{\mathrm{N}}_{\mathrm{A}}v\left( {\rho - \rho _0} \right)\left( {H_1 - H_2} \right){\mathrm{g}}}}{{{\mathrm{R}}T}}}$$

where the *c*_1_ and *c*_2_ are the colloidal concentration at the height of *H*_1_ and *H*_2_; the N_A_ is the Avogadro constant; the *v* is the volume of the colloidal particle; the *ρ* and *ρ*_0_ denote the density of the colloidal particles and the solvent, respectively; the g is gravitational acceleration constant; the R is constant and the *T* is the temperature.

Figure 2a shows the dynamic light scattering (DLS) results of MAI_x_-BAI_y_-PbI_2_ precursors in the solvents. We take the MAI-PbI_2_ (the mole ratio is 1:1, presenting the *n* is infinity) and BAI-PbI_2_ (the mole ratio is 2:1, presenting the *n* is 1) as the example. As shown in Fig. 2a, the average colloidal sizes of the MAI-PbI_2_ clusters and BAI-PbI_2_ cluster are about 180 nm and 320 nm, respectively. Thus, at a certain height of *H*_1_ and *H*_2_ (where *H*_1_ > *H*_2_, and *H*_2_ is near the substrate), since BAI-PbI_2_ colloid has a larger volume than MAI-PbI_2_ colloid (*v*_BA_ > *v*_MA_,), the difference in BAI-PbI_2_ concentration at the height of *H*_2_ and *H*_1_ [denoted as $( {\frac{{c_2}}{{c_1}}} )_{{\mathrm{BA}}}$] and that of MAI-PbI_2_ [${\mathrm{denoted}}\,{\mathrm{as}}\,( {\frac{{c_2}}{{c_1}}} )_{{\mathrm{MA}}}$] will meet the equation: $( {\frac{{c_2}}{{c_1}}} )_{{\mathrm{BA}}} > ( {\frac{{c_2}}{{c_1}}} )_{{\mathrm{MA}}}$, which can be transformed into $\frac{{\left( {c_2} \right)_{{\mathrm{BA}}}}}{{\left( {c_2} \right)_{{\mathrm{MA}}}}} > \frac{{\left( {c_1} \right)_{{\mathrm{BA}}}}}{{\left( {c_1} \right)_{{\mathrm{MA}}}}}$. Therefore, the mole ratio of BAI/MAI at the bottom is larger than that at the top. This explains the phenomenon generally observed in Q-2D perovskite films that small-*n* phases locate at the bottom of the perovskite films while large-*n* phases locate at the upper surface of the perovskite films when deposited on glass substrates.

However, when deposited on the PEDOT:PSS, the vertical distribution of *n* from the small (bottom side) to the large (top side) disappears in the Q-2D perovskite films. The rule of sedimentation equilibrium can’t explain the observation of the Q-2D perovskite films deposited on the PEDOT:PSS. First we consider the possible effect of surface energy. Contact angle on glass substrate (43.48°, Supplementary Figure 4) which is similar to that of BrB-PEDOT:PSS (46.79°). Considering that their PL spectra are quite different, the surface energy is not the main reason of the vertical *n* distribution. Then, we hypothesize the change of the vertical distribution of *n* is associated to the change of the precursor solutions. The PSSH acid is soluble in the processing solvent of the perovskite (DMSO)^47,48^. As shown in the Supplementary Figure 5, the XPS spectra show the removal of PSSH. After the spin-coating of pure DMSO on PEDOT:PSS, the intensity of the sulfur atoms in PSSH (binding energy between 166 and 172 eV) decreases, while the intensity of the sulfur atoms in PEDOT is unchanged (binging energy between 162 and 166 eV). Previously, it has been reported that the acid addition will reduce the colloid concentration in the perovskite precursors^49–51^. Because the PSSH itself is in colloid formation (the Tyndall effect of PSSH solution is shown in Supplementary Figure 6), we added HI instead of PSSH to study the effect. As shown in Fig. 2a, upon the addition of HI (3 vol.%), the large-sized colloids disappear and the precursor becomes solution, which is accordance with the previous study^49,50^. The photographs of the Q-2D perovskite precursor with and without acid addition were shown in Fig. 2b, c, respectively. After the addition of the acid, light scattering can hardly be observed (Fig. 2c). To certify that the acid addition suppresses the vertical phase distribution, we deposited the Q-2D perovskite precursor with the addition of HI, PSSH, and water on glass substrates and measured their PL from the back side (glass side). As shown in Fig. 2d, the peaks of small-*n* phases are much less pronounced when the HI or PSSH acid is added into the precursor.

Furthermore, we deposited Q-2D perovskite films on PEDOT:PSS, PSSH and DMSO treated PEDOT:PSS and measured their PL from the back side. The DMSO treatment denotes the spin coating of pure DMSO on top of the PEDOT:PSS film. As shown in Supplementary Figure 7, the peaks of small-*n* phases are stronger on DMSO treated PEDOT:PSS surface than the films on the other two surface because of the fewer PSSH. After BrB-modification, the PSSH was converted into PSS-BrB and the ionic bond was converted into covalent bond. The non-acid PEDOT:PSS surface after BrB modification will maintain the colloidal property of the precursors. Therefore, the vertical distribution of *n* can be obtained when the Q-2D perovskite is deposited on the BrB-PEDOT:PSS. Figure 2e demonstrates the schematic of phase distribution in Q-2D films deposited on PEDOT:PSS and BrB-PEDOT:PSS substrates.

### Morphology and crystallinity of the Q-2D perovskite films

**Fig. 3:**
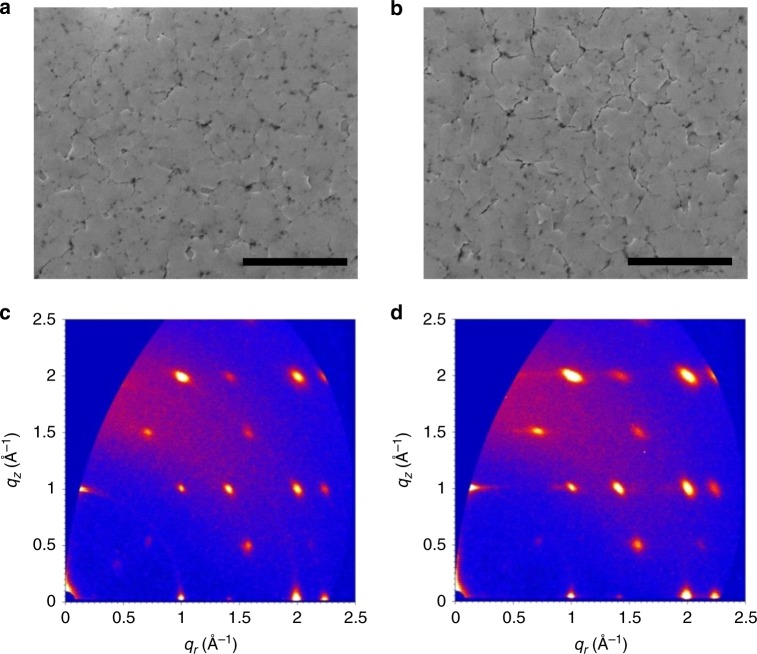
Morphology and crystallinity of the Q-2D perovskite films. SEM images of Q-2D perovskite films on: **a** PEDOT:PSS and **b** BrB-PEDOT:PSS (the scale bar is 4 μm). GIWAXS scatting pattern of Q-2D perovskite films on: **c** PEDOT:PSS and **d** BrB-PEDOT:PSS

We also characterize the morphology and the crystallinity with scanning electron microscopy (SEM) and grazing incidence wide-angle X-ray scattering (GIWAXS) to investigate the perovskite films on different HTLs. From the SEM images shown in Fig. 3a, b, both the films show the uniform and compact coverage. Besides, both the GIWAXS patterns demonstrate discrete Bragg spots, indicating highly orientated crystalline (shown in Fig. 3c, d). Moreover, the orientated structure is beneficial for efficient charge carrier transport^29^. The Q-2D perovskite films on BrB-PEDOT:PSS show the same set of diffraction spots as that on PEDOT:PSS substrate, but stronger intensity. The GIWAXS intensity profiles integrated over a polar angle from 0 to 90 degree are shown in the Supplementary Figure 8. The higher crystallinity can be attributed to the more hydrophobic substrate of BrB-PEDOT:PSS^52^.

### Photovoltaic performance of devices

**Fig. 4:**
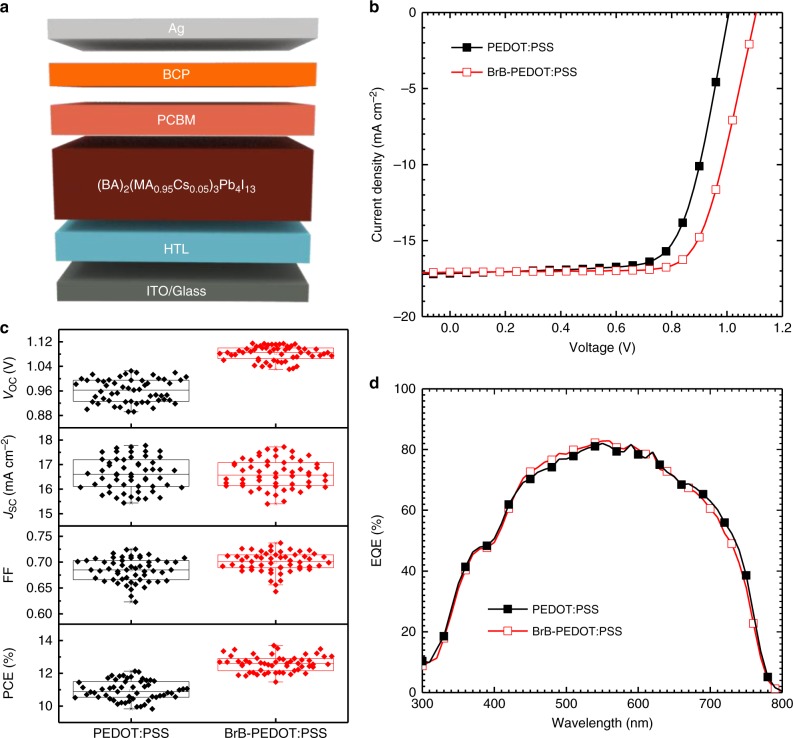
Photovoltaic performance of devices with tailored vertical distribution. **a** Device structure of the Q-2D perovskite solar cells; **b** *J–V* characteristics of the cells with PEDOT:PSS and BrB-PEDOT:PSS; **c** Photovoltaic parameters of 55 devices for each HTL; (Boxes, square symbols, and horizontal bars indicate 25/75 percentile, mean, and min/max values, respectively). **d** EQE spectra of Q-2D perovskite solar cells with different PEDOT:PSS and BrB-PEDOT:PSS HTLs

We fabricated photovoltaic devices in the planar invert structure of ITO/HTL/(BA)_2_(MA_0.95_Cs_0.05_)_3_Pb_4_I_13_/PCBM/BCP/Ag (where the HTL is PEDOT:PSS or BrB-PEDOT:PSS) and measured devices under the AM 1.5G solar simulator. The device structure and the current density–voltage (*J–V*) curves of devices are presented in Fig. 4a, b. Reference devices with PEDOT:PSS HTL delivered an champion PCE of 12.29% where open-circuit voltage (*V*_OC_), short-circuit current density (*J*_SC_), and fill factor (FF) are 1.01 V, 17.16 mA cm^−2^, and 0.71, respectively. The performance is comparable to those reported in the literature of devices with the similar structure^36^. With the BrB-PEDOT:PSS as HTL, the PCE of the cells reaches 13.74% with an significant improvement of *V*_OC_ (1.11 V). To the best of our knowledge, this is the highest *V*_OC_ regarding BA-based Q-2D (*n* = 4, derived from the precursor formulation) based perovskite solar cell. Supplementary Table 1 summarized the previously reported BA-based Q-2D perovskite solar cell performance. The statistical data of 55 devices are summarized in Fig. 4c and Table 1. The *V*_OC_ increases from 0.96 ± 0.04 to 1.08 ± 0.02 V and the FF slightly increases from 0.68 ± 0.02 to 0.70 ± 0.02 while the *J*_SC_ nearly stays the same. Moreover, the BrB-PEDOT:PSS based devices have the narrower distribution of *V*_OC_. The *J*_SC_ was confirmed by the integration of the EQE spectrum (shown in Fig. 4d). The devices show negligible hysteresis under different scan directions and different scan rates (shown in Supplementary Figure 9 and 10). The stability efficiency at the maximum power point is shown in the Supplementary Figure 11. Performance of devices processed from other conditions (using pure GBL solvent to process the perovskite layer, or the perovskite layer without Cs addition) is shown in Supplementary Figure 12 and 13, respectively. They both show inferior performance (8.6 and 10.9% after BrB modification, respectively) than the optimized devices with 5% Cs addition and processed from GBL:DMSO mixed solvent (13.74%).

**Table 1:** Photovoltaic parameters of Q-2D perovskite solar cells with PEDOT:PSS and BrB-PEDOT:PSS HTLs

HTL		*V*_OC_ (V)	*J*_SC_ (mA cm^−2^)	*J*_SC,EQE_ (mA cm^−2^)^b^	FF (%)	PCE (%)
PEDOT:PSS	Average	0.96 ± 0.04	16.66 ± 0.62		68.5 ± 2.29	10.96 ± 0.60
	Best^a^	1.01	17.16	16.73	70.9	12.29
BrB-PEDOT:PSS	Average	1.08 ± 0.02	16.62 ± 0.57		70.0 ± 1.92	12.59 ± 0.50
	Best^a^	1.11	17.08	16.60	72.5	13.74

^a^The highest efficiency

^b^*J*_SC, EQE_ represents the integrated current density obtained from EQE spectra

It should be noted that we have also applied the BrB-PEDOT:PSS as HTL in conventional 3D perovskite solar cells. There is no such significant enhancement in *V*_OC_ (shown in Supplementary Figure 14). The *V*_OC_ is 0.93 and 0.91 V for the 3D perovskite solar cells with BrB-PEDOT:PSS and PEDOT:PSS HTLs, respectively. This also confirms the important role of the BrB-PEDOT:PSS tailoring the vertical phase distribution in Q-2D perovskite films. In 3D perovskite films, there is no such tailoring of *n* values. The *n* value is infinite throughout the entire 3D perovskite films.

### Charge recombination and dynamics

**Fig. 5:**
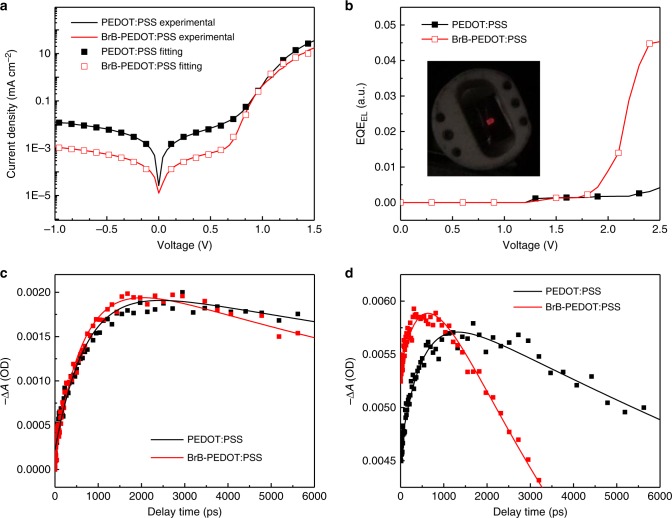
Charge recombination and dynamics of the Q-2D perovskite films with tailored vertical distribution. **a** *J–V* characteristics of the devices with PEDOT:PSS and BrB-PEDOT:PSS measured in the dark and their fit with one-diode equivalent circuit model; **b** EQE_EL_ as a function of voltage of the devices with PEDOT:PSS and BrB-PEDOT:PSS HTL (functioning as light-emitting diodes). The inset shows EL emission of a device with BrB-PEDOT:PSS HTL; **c** TA kinetics probed at 740 nm under back-excitation at 480 nm, showing the electron transfer kinetics; **d** TA kinetics probed at 605 nm under front-excitation at 740 nm, showing the hole transfer kinetics

The improvement of the solar cell performance after the BrB modification on PEDOT:PSS is mainly ascribed to the increase of *V*_OC_ from 0.96 ± 0.04 to 1.08 ± 0.02 V. Based on the expression of *V*_OC_ = *n*_if_K*T/*qln(*J*_SC_/*J*_sat_) (where K is Boltzmann’s constant, *T* is the temperature, *n*_if_ is the diode ideality factor, *J*_sat_ is the dark saturation current and q is elementary charge), smaller *J*_sat_ that suggest lower recombination will result in higher *V*_OC_^53,54^. The *J*_sat_ and *n*_if_ values are extracted from dark *J–V* characteristics fitted with one-diode equivalent circuit model (Fig. 5a). The resulting parameters are summarized in Supplementary Table 2. The *J*_sat_ was fitted to 1.33 × 10^−9^ mA cm^−2^ (cell with BrB-PEDOT) and 1.41 × 10^−7^ mA cm^−2^ for PEDOT:PSS (cell with PEDOT:PSS), and the *n*_if_ were fitted to 1.89 (cell with BrB-PEDOT) and 2.22 (cell with PEDOT:PSS). The calculated *V*_OC_ is 1.06 V for PEDOT:PSS based devices and 1.15 V for BrB-PEDOT:PSS based devices.

*V*_OC_ loss by non-radiative recombination (Δ*V*_OC,non-rad_) of perovskite solar cells is evaluated with the expression: Δ*V*_OC,non-rad_ = −K*T/*qln(EQE_EL_)^55–57^, where EQE_EL_ is the electroluminescence (EL) EQE of cells working as light-emitting diodes. We measured the EL of the both cells with PEDOT:PSS and BrB-PEDOT:PS HTL. When the injection current is equal to the *J*_SC_ at about 1.5 V, the luminescent intensity is below the detection limit of the photodiode. At the applied voltage over 1.8 V, the EQE_EL_ of the cell with BrB-PEDOT:PSS is higher than that of the cell with PEDOT:PSS (Fig. 5b). The EL spectra of the two cells are shown in the Supplementary Figure 15. The higher EQE_EL_ suggests lower *V*_OC_ loss by the non-radiative recombination for the cell with BrB-HTL.

Trap density is important to the non-radiative recombination and the *V*_OC_^58^. Here, we evaluate the trap-state density using space charge limited current (SCLC) method by fabricating the hole-only devices (ITO/HTL/Q-2D/spiro-OMeTAD/Au, where the HTL is PEDOT:PSS or BrB-PEDOT:PSS). The trap-state density was calculated according to the equation:

2 $$N_{\mathrm{t}} = \frac{{2{\mathrm{\varepsilon }}_{\mathrm{o}}{{\varepsilon}}_{\mathrm{r}}V_{\mathrm{TFL}}}}{{{\mathrm{q}}L^2}}$$

where ε_o_ and ε_r_ are the dielectric constants of vacuum and the perovskite, *V*_TFL_ is the threshold voltage of trap-filled limit (TFL) region, q is the elementary charge, and *L* is the thickness of the film (about 400 nm). From the current–voltage curves shown in Supplementary Figure 16a, we can calculate that the trap density of BrB-PEDOT:PSS based Q-2D films is about (1.02 ± 0.18) × 10^16^ cm^−3^ which is lower than that of PEDOT:PSS based Q-2D films ((1.47 ± 0.14) × 10^16^ cm^−3^). A slightly hysteresis can be observed in the SCLC measurement (Supplementary Figure 16b and 16c), and the *V*_TFL_ values are similar in both scanning directions that derive similar trap densities.

To explore the influence of vertical phase distribution on the charge transport, ultrafast transient absorption (TA) measurement was performed to reveal the carrier dynamics. Supplementary Figure 17 shows the TA spectra at different delay time of the Q-2D perovskite films (about 400 nm thick) excited from the back side (glass side) at 480 nm, 1 μJ cm^−2^ per pulse. The TA spectra show several bleach peaks which agree with the previous work^38,39^. For BrB-PEDOT:PSS based Q-2D films, the bleach peak at 600 nm is stronger than that of PEDOT:PSS based Q-2D films. This also indicates more small-*n* phases exist at the substrate side when the substrate is BrB-PEDOT:PSS. The bleach recovery of the small-*n* phases and the generation of the large *n* phase (at 740 nm) are the proofs of electron transport from the small-*n* to large-*n* phases^38^. The TA kinetics probed at 740 nm is fitted as a function of time with bi-exponential function shown in Fig. 5c (fitting parameters are in Supplementary Table 3). In term of hole transfer, we measured the TA spectra on the Q-2D perovskite film excited from the front side (top side) at 740 nm, 10 μJ cm^−2^ per pulse. At this excitation wavelength, only the large-*n* phase can be excited. However, besides the broad photoinduced absorption signal from large *n*, small-*n* (*n* = 3 perovskite phases) bleach peak is also observed (Supplementary Figure 18), owing to the hole transport from large-*n* to small-*n* phases. TA kinetics at 605 nm (Fig. 5d, after subtracting the photoinduced absorption contribution) is fitted to calculate the hole transport time from top to bottom (the fitting parameters are shown in Supplementary Table 4). When the substrate surface is BrB-PEDOT:PSS, both the electron and hole transfers in Q-2D perovskite films are faster compared to those in reference films on PEDOT:PSS films (the rising part shown in Fig. 5c, d). The bleach recovery kinetics in Fig. 5c, d is attributed partially to the carrier extraction^59^. The shorter delay time of the Q-2D perovskite films on BrB-PEDOT:PSS indicates that charge extraction occurs more efficiently in the case of BrB-PEDOT:PSS.

Supplementary Figure 19 shows the device performance evolution of the Q-2D perovskite solar cells stored in the dark in air without encapsulation. After kept in air for 1400 h, both Q-2D devices show a good stability. The PCEs remain 80% and 88% of their initial values for cells with PEDOT:PSS and BrB-PEDOT:PSS HTL, respectively. The main loss is due to the FF which might come from the degeneration between the interfaces of the Q-2D perovskite films and the electrodes. The better stability of the device with BrB-PEDOT:PSS HTL is probably attributed to the more hydrophobic property of BrB-PEDOT:PSS^60^.

## Discussion

Vertical phase (layer numbers, *n*) distribution in Q-2D perovskite films has been observed^38^, but the driving force of vertical *n* distribution has been not discussed or reported. In this work, we have reported that the vertical phase distribution of Q-2D perovskite films on glass substrates can be explained by the sedimentation equilibrium because of the colloidal feature of the precursors, and can be tailored by the surface modification and acid addition. Main observations and conclusions include: (1) the vertical phase distribution of Q-2D perovskite films on PEDOT:PSS HTL is different from the glass substrates. More small-*n* phases in the Q-2D perovskite films form at the bottom side (close to substrate) when deposited on glass substrates, compared to the Q-2D perovskite films deposited on PEDOT:PSS layers; (2) The origin of the vertical phase distribution of *n* is the colloid property of the perovskite precursors (the colloid size on the order of 100–1000 nm) that follows the sedimentation equilibrium. Once the colloid properties of precursors are changed to solution, the vertical distribution with different *n* will not observed any more. The addition of the acid into the perovskite precursors will change the precursors from the colloid to solution; (3) For the surface of PEDOT:PSS, when the perovskite is deposited on top of it, the acid component of PSSH will be dissolved and changes colloid properties of the precursors into solution. The vertical distribution with different *n* can hardly be observed in the Q-2D perovskite films when deposited on PEDOT:PSS. A 4-bromobenzenediazonium (BrB) tetrafluoroborate covalently anchored onto the surface of the PEDOT:PSS can mitigate the change of the colloid properties of the precursors, and therefore induces the formation of small-*n* phase close to the BrB-PEDOT:PSS surface in the Q-2D perovskite.

Vertical phase distribution in the Q-2D perovskite films is critical to the carrier dynamics and device performance. The tailored distribution with BrB modification is favorable for charge dissociation and transport. A high *V*_OC_ of 1.11 V and a PCE of 13.74% are achieved in the Q-2D perovskite solar cells with the BrB-PEDOT:PSS as the HTL. Furthermore, the more hydrophobic surface of the BrB-surface anchoring also enhances the device stability. The report here will also intrigue the community to notice the importance and develop strategies for energetically favorable vertical phase distribution in Q-2D perovskite films for solar cells as well as light-emitting diode applications.

## Methods

### Device and sample fabrication

PEDOT:PSS (Clevios P VP AI 4083) was deposited on the cleaned ITO (8 ohm sq^−1^) by spin-coating at 4000 rpm for 40 s and annealed at 130 °C for 15 min. 4-bromobenzenediazonium tetrafluoroborate (97% TCI, 0.2 mg mL^−1^ in H_2_O) was deposited on the PEDOT:PSS by spin coating and washed by isopropanol. After annealed at 100 °C for 10 min, the BrB-PEDOT:PSS HTL was obtained. The Q-2D perovskite was deposited onto pre-heated substrates (100 °C) by spin coating at 4000 rpm for 30 s, followed by thermal annealing at 100 °C for 10 min. The perovskite precursor was comprised of CsI (99.99%, Xi’an Polymer Light Technology Corp), MAI (99.5%, Xi’an Polymer Light Technology Corp), BAI (99.5%, Xi’an Polymer Light Technology Corp) and PbI_2_ (99.9875%, Alfa Aesar) with mole ratio at 0.15:2.85:2:4 in mixed solvents of GBL:DMSO (7:3, volume ratio) with the Pb^2+^ concentration of 1 M. The precursor was stirring at 60 °C for 2 h. PCBM (99 %, Xi’an Polymer Light Technology Corp, 15 mg mL^−1^ in chloroform) was deposited on Q-2D perovskite by spin coating at 3000 rpm for 40 s and BCP (98 %, Alfa Aesar, 0.5 mg mL^−1^ in isopropanol) was deposited on top of the PCBM layer by spin coating at 5000 rpm for 40 s. Finally, 120 nm silver electrodes were evaporated under high vacuum (below 2 × 10^−7^ Torr) through a shadow mask using a thermal evaporator (Mini-spectros, Kurt J. Lesker). The effective active area of the devices is 4.1 mm^2^.

For optical (absorption and PL) and morphology (SEM, GIWAXS) characterization, the perovskite samples were deposited on different substrates (glass, PEDOT:PSS, BrB-PEDOT:PSS) via the hot-casting method that is the same as that used for the devices.

### Characterization

The current *J–V* curves of the cells were measured using a Keithley 2400 SourceMeter. The cells were illuminated through an aperture area of 4.095 mm^2^ from a 100 mW cm^−2^ AM1.5 solar simulator (Newport, ORIEL, Sol3A, 450 W xenon lamp). The area of the aperture is confirmed by the National Institute of Metrology (NIM, Beijing). There is nearly no variation of the performance with or without aperture. The measurement was performed in a N_2_-fillerd glove box at the temperature of 25 °C. Light intensity of the light source was calibrated with a silicon photodiode (Hamamatsu, S1133-01) which has been certified by National Renewable Energy Laboratory (NREL). The scan rate was 0.2 V s^−1^ and the dwell time was 0.1 s.

The external quantum efficiency (EQE) test was characterized using a 150 W xenon lamp (Oriel) filtered with a monochromator (Cornerstone 74004) as a monochromatic light source.

The EL measurement was performed on a home-built system. A Si photodiode (FU-G010, the area is 1 cm^2^) was placed on back of the device to record the EL intensity. A Keithley 2400 SourceMeter is used to apply the voltage and collect the current. The other 2400 SourceMeter was used to measure the current of the Si photodiode. The EQE_EL_ was expected to be underestimated because angular dependence of emission and the detector sensitivity were not considered.

SEM images were conducted by a high-resolution field emission scanning electron microscope (FEI Nova Nano-SEM 450). The absorbance of Q-2D perovskite films was acquired by a UV-vis-NIR Spectrophotometer (UV-3600, Shimadzu). FT-IR experiments were performed on the VERTEX 70, Bruker. The PL measurements were performed using a fluorescence spectrometer (Edinburgh Instruments FLS920) with a 150 W Xe lamp as an excitation source at 450 nm. In-depth XPS (AXIS-ULTRA DLD-600W) profiling was performed on the samples of glass/HTL/Q-2D, where the HTL is PEDOT:PSS or BrB-PEDOT:PSS. The samples were etched by Ar_2_. The C and Pb atom ratios were detected. The femtosecond TA setup is based on a regenerative amplified Ti:sapphire laser system from Coherent (800 nm, 35 fs, 6 mJ pulse, and 1 kHz repetition rate), nonlinear frequency mixing techniques and the ultrafast TA spectrometer (Time-Tech Spectra, femtoTA-100). GIWAXS measurements were carried out with a Xeuss 2.0 SAXS/WAXS laboratory beamline using a Cu X-ray source (8.05 keV, 1.54 Å) and a Pilatus3R 300K detector. The incidence angle is 0.3°.

The contact angle was measured via an optical video contact angle instrument (VCA Optima XE, AST) at room temperature and was determined after a water droplet placed on the sample. Zeta potential and the dynamic light scattering measurement were tested on a Malvern Zetasizer Naono ZS. Concentration for the two measurements of the samples is 1 mg mL^−1^.

## Supplementary information

Supplementary Information

Source Data

## Data Availability

The data that support the findings of this study are available from the corresponding author upon reasonable request. Source data of Figs. 1a, 1b, 1f, 2a, 2d, 4b–d, 5 and Supplementary Figures 2, 3, 5, 7-19 are provided as a Source Data file.

## References

[1] De Wolf S (2014). Organometallic halide perovskites: sharp optical absorption edge and its relation to photovoltaic performance. J. Phys. Chem. Lett..

[2] Miyata A (2015). Direct measurement of the exciton binding energy and effective masses for charge carriers in organic-inorganic tri-halide perovskites. Nat. Phys..

[3] Dong Q (2015). Solar cells. Electron-hole diffusion lengths>175 mum in solution-grown CH3NH3PbI3 single crystals. Science.

[4] Stranks SD (2013). Electron-hole diffusion lengths exceeding 1 micrometer in an organometal trihalide perovskite absorber. Science.

[5] Xing G (2013). Long-range balanced electron- and hole-transport lengths in organic-inorganic CH3NH3PbI3. Science.

[6] Shi D (2015). Solar cells. Low trap-state density and long carrier diffusion in organolead trihalide perovskite single crystals. Science.

[7] Yin WJ, Shi TT, Yan YF (2014). Unique properties of halide perovskites as possible origins of the superior solar cell performance. Adv. Mater..

[8] Weidman MC, Seitz M, Stranks SD, Tisdale WA (2016). Highly tunable colloidal perovskite nanoplatelets through variable cation, metal, and halide composition. ACS Nano.

[9] Noh JH, Im SH, Heo JH, Mandal TN, Seok SI (2013). Chemical management for colorful, efficient, and stable inorganic-organic hybrid nanostructured solar cells. Nano Lett..

[10] National Renewable Energy Laboratory. Best Research-Cell Efficiencies Chart. https://www.nrel.gov/pv/assets/images/efficiency-chart.png, 2018.

[11] Niu GD, Guo XD, Wang LD (2015). Review of recent progress in chemical stability of perovskite solar cells. J. Mater. Chem. A.

[12] Leijtens T (2015). Stability of metal halide perovskite solar cells. Adv. Energy Mater..

[13] Park NG, Gratzel M, Miyasaka T, Zhu K, Emery K (2016). Towards stable and commercially available perovskite solar cells. Nat. Energy.

[14] Zhao X, Park NG (2015). Stability issues on perovskite solar cells. Photonics.

[15] Yang J, Siempelkamp BD, Liu D, Kelly TL (2015). Investigation of CH3NH3PbI3 degradation rates and mechanisms in controlled humidity environments using in situ techniques. ACS Nano.

[16] Li FM, Liu MZ (2017). Recent efficient strategies for improving the moisture stability of perovskite solar cells. J. Mater. Chem. A.

[17] Dou L (2015). Atomically thin two-dimensional organic-inorganic hybrid perovskites. Science.

[18] Wang NN (2016). Perovskite light-emitting diodes based on solution-processed self-organized multiple quantum wells. Nat. Photonics.

[19] Wang Z (2017). Efficient ambient-air-stable solar cells with 2D–3D heterostructured butylammonium-caesium-formamidinium lead halide perovskites. Nat. Energy.

[20] Yan J, Qiu W, Wu G, Heremans P, Chen H (2018). Recent progress in 2D/quasi-2D layered metal halide perovskites for solar cells. J. Mater. Chem. A.

[21] Cao DH, Stoumpos CC, Farha OK, Hupp JT, Kanatzidis MG (2015). 2D homologous perovskites as light-absorbing materials for solar cell applications. J. Am. Chem. Soc..

[22] Lermer C (2016). Toward fluorinated spacers for MAPI-derived hybrid perovskites: synthesis, characterization, and phase transitions of (FC2H4NH3)2PbCl4. Chem. Mater..

[23] Smith IC, Hoke ET, Solis-Ibarra D, McGehee MD, Karunadasa HI (2014). A layered hybrid perovskite solar-cell absorber with enhanced moisture stability. Angew. Chem. Int. Ed..

[24] Quan LN (2016). Ligand-stabilized reduced-dimensionality perovskites. J. Am. Chem. Soc..

[25] Stoumpos CC (2016). Ruddlesden-Popper hybrid lead iodide perovskite 2D homologous semiconductors. Chem. Mater..

[26] Tsai H (2018). Design principles for electronic charge transport in solution-processed vertically stacked 2D perovskite quantum wells. Nat. Commun..

[27] Chen Y (2018). 2D Ruddlesden-Popper perovskites for optoelectronics. Adv. Mater..

[28] Chen YN (2017). Tailoring organic cation of 2D air-stable organometal halide perovskites for highly efficient planar solar cells. Adv. Energy Mater..

[29] Tsai HH (2016). High-efficiency two-dimensional Ruddlesden-Popper perovskite solar cells. Nature.

[30] Soe CMM (2018). Understanding film formation morphology and orientation in high member 2D Ruddlesden-Popper perovskites for high-efficiency solar cells. Adv. Energy Mater..

[31] Chen AZ (2018). Origin of vertical orientation in two-dimensional metal halide perovskites and its effect on photovoltaic performance. Nat. Commun..

[32] Zhang X (2018). Phase transition control for high performance Ruddlesden-Popper perovskite solar cells. Adv. Mater..

[33] Zhang XQ (2018). Orientation regulation of phenylethylammonium cation based 2D Perovskite solar cell with efficiency higher than 11%. Adv. Energy Mater..

[34] Zhang X (2017). Vertically oriented 2D layered perovskite solar cells with enhanced efficiency and good stability. Small.

[35] Qing J (2018). Aligned and graded type-II Ruddlesden-Popper perovskite films for efficient solar cells. Adv. Energy Mater..

[36] Zhou N (2018). Exploration of crystallization kinetics in quasi two-dimensional perovskite and high performance solar cells. J. Am. Chem. Soc..

[37] Zhang X (2017). Stable high efficiency two-dimensional perovskite solar cells via cesium doping. Energy Environ. Sci..

[38] Liu J, Leng J, Wu K, Zhang J, Jin S (2017). Observation of internal photoinduced electron and hole separation in hybrid two-dimentional perovskite films. J. Am. Chem. Soc..

[39] Shang Q (2017). Unveiling structurally engineered carrier dynamics in hybrid quasi-two-dimensional perovskite thin films toward controllable emission. J. Phys. Chem. Lett..

[40] Li L (2018). Unraveling the growth of hierarchical quasi-2D/3D perovskite and carrier dynamics. J. Phys. Chem. Lett..

[41] Zheng KB (2018). Inter-phase charge and energy transfer in Ruddlesden-Popper 2D perovskites: critical role of the spacing cations. J. Mater. Chem. A.

[42] Guselnikova OA (2017). Tuning of PEDOT: PSS properties through covalent surface modification. J. Polym. Sci. Pol. Phys..

[43] Xiong Z, Gu T, Wang X (2014). Self-assembled multilayer films of sulfonated graphene and polystyrene-based diazonium salt as photo-cross-linkable supercapacitor electrodes. Langmuir.

[44] Yan K (2015). Hybrid halide perovskite solar cell precursors: colloidal chemistry and coordination engineering behind device processing for high efficiency. J. Am. Chem. Soc..

[45] Li B, Li M, Fei C, Cao G, Tian J (2017). Colloidal engineering for monolayer CH3NH3PbI3 films toward high performance perovskite solar cells. J. Mater. Chem. A.

[46] Johnston N, Howell LG (1930). Sedimentation equilibria of colloidal particles. Phys. Rev..

[47] Lee SH (2014). Modified physico-chemical properties and supercapacitive performance via DMSO inducement to PEDOT: PSS active layer. Org. Electron..

[48] Xia YJ, Sun K, Chang JJ, Ouyang JY (2015). Effects of organic inorganic hybrid perovskite materials on the electronic properties and morphology of poly(3,4-ethylenedioxythiophene):poly(styrenesulfonate) and the photovoltaic performance of planar perovskite solar cells. J. Mater. Chem. A.

[49] McMeekin DP (2017). Crystallization kinetics and morphology control of formamidinium–cesium mixed-cation lead mixed-halide perovskite via tunability of the colloidal precursor solution. Adv. Mater..

[50] Noel NK (2017). Unveiling the influence of pH on the crystallization of hybrid perovskites, delivering low voltage loss photovoltaics. Joule.

[51] Nayak PK (2016). Mechanism for rapid growth of organic–inorganic halide perovskite crystals. Nat. Commun..

[52] Bi C (2015). Non-wetting surface-driven high-aspect-ratio crystalline grain growth for efficient hybrid perovskite solar cells. Nat. Commun..

[53] You J (2014). Moisture assisted perovskite film growth for high performance solar cells. Appl. Phys. Lett..

[54] Jiang Y (2016). Enhancement of photovoltaic performance by utilizing readily accessible hole transporting layer of vanadium(V) oxide hydrate in a polymer–fullerene blend solar cell. ACS Appl. Mater. Interfaces.

[55] Bi D (2016). Efficient luminescent solar cells based on tailored mixed-cation perovskites. Sci. Adv..

[56] Saliba M (2016). Incorporation of rubidium cations into perovskite solar cells improves photovoltaic performance. Science.

[57] Yao J (2015). Quantifying losses in open-circuit voltage in solution-processable solar cells. Phys. Rev. Appl..

[58] Shao YC, Yuan YB, Huang JS (2016). Correlation of energy disorder and open-circuit voltage in hybrid perovskite solar cells. Nat. Energy.

[59] Leng J, Liu J, Zhang J, Jin S (2016). Decoupling interfacial charge transfer from bulk diffusion unravels its intrinsic role for efficient charge extraction in perovskite solar cells. J. Phys. Chem. Lett..

[60] Liu T (2016). Nonreduction-active hole-transporting layers enhancing open-circuit voltage and efficiency of planar perovskite solar cells. ACS Appl. Mater. Interfaces.

