# Supplementary material for: Tailoring vertical phase distribution of quasi-two-dimensional perovskite films via surface modification of hole-transporting layer
Source: Nat Commun. 2019 Feb 20;10:878. doi: 10.1038/s41467-019-08843-5 (PMC6382759; doi:10.1038/s41467-019-08843-5)
Supplement: Supplementary file 1 — Supplementary Information [file 41467_2019_8843_MOESM1_ESM.pdf]

Supplementary information

Tailoring vertical phase distribution of quasi two-dimensional perovskite films via surface modification of hole-transporting layer

Liu et al.

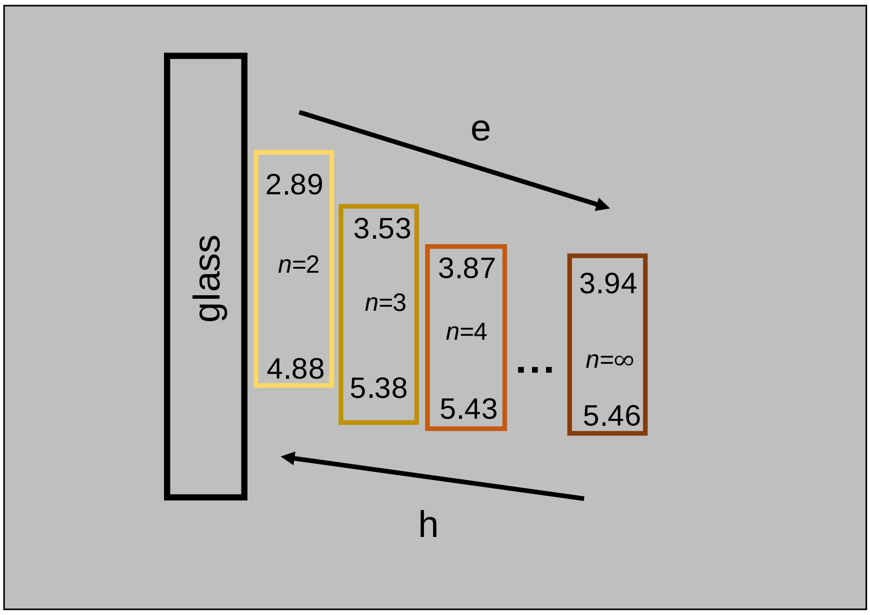

Supplementary Figure 1. Energy level diagram of Q-2D perovskite with the *n* increasing from 2 to infinity (3D). The energy level values are extracted from the previous work reported by Kanatzidis.[1]

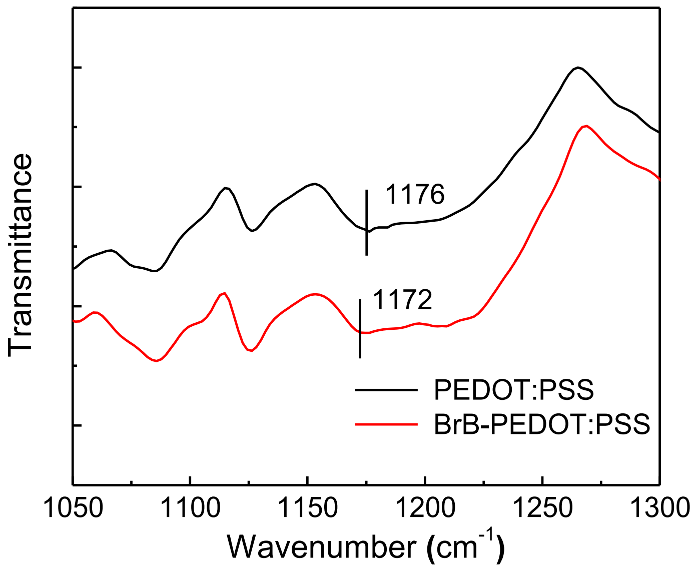

Supplementary Figure 2. FT-IR spectra of the PEDOT:PSS and BrB-PEDOT:PSS films that indicate the conversion from ionic bond ($-SO_3^-$) to covalent bond ($-SO_2-O-$).

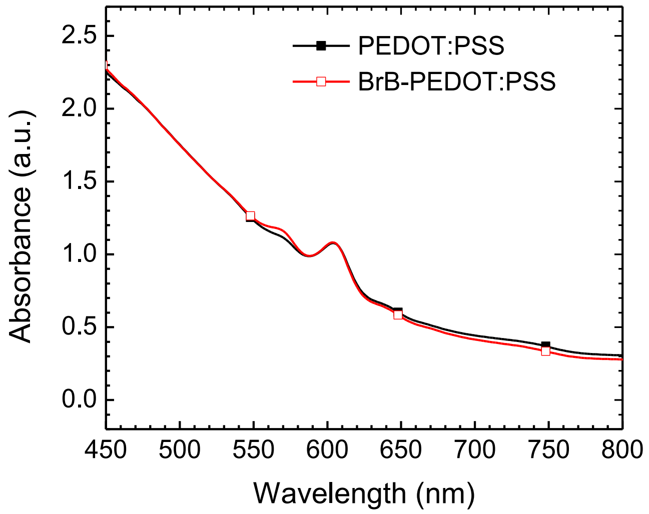

Supplementary Figure 3. Absorbance spectra of the Q-2D perovskite films on PEDOT:PSS or BrB-PEDOT:PSS.

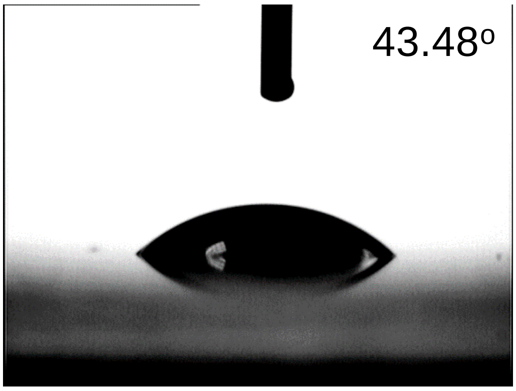

Supplementary Figure 4. Contact angle images of water on glass substrate.

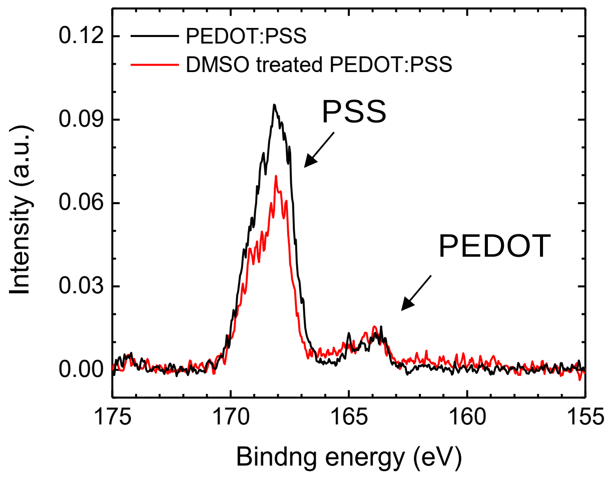

Supplementary Figure 5. XPS spectra of PEDOT:PSS film and DMSO treated PEDOT:PSS film. The DMSO treatment denotes the spin coating of DMSO on top of the PEDOT:PSS film.

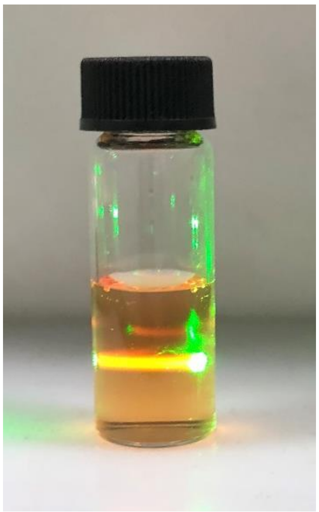

Supplementary Figure 6. Picture showing the Tyndall effect (light scattering) in the 5 wt.% PSSH solution.

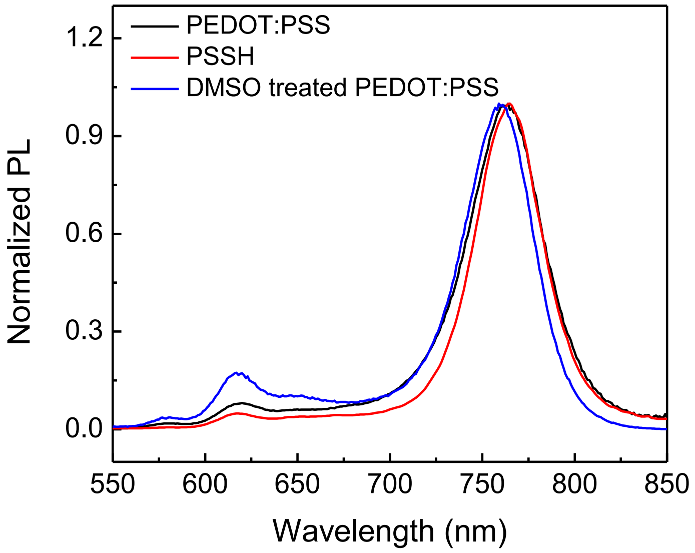

Supplementary Figure 7. PL spectra of Q-2D perovskite films excited from the back side (from the glass side) deposited on the PEDOT:PSS, PSSH and DMSO treated PEDOT:PSS. The DMSO treatment denotes the spin coating of pure DMSO on top of the PEDOT:PSS film.

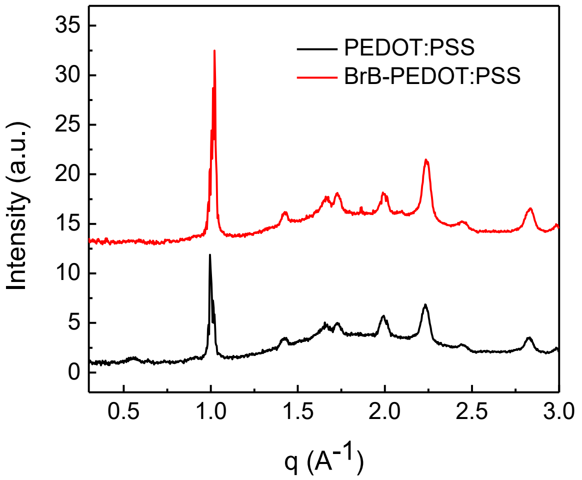

Supplementary Figure 8. Integrated intensity from the GIWAXS measurement results of Q-2D films along the sector on the PEDOT:PSS and BrB-PEDOT:PSS films.

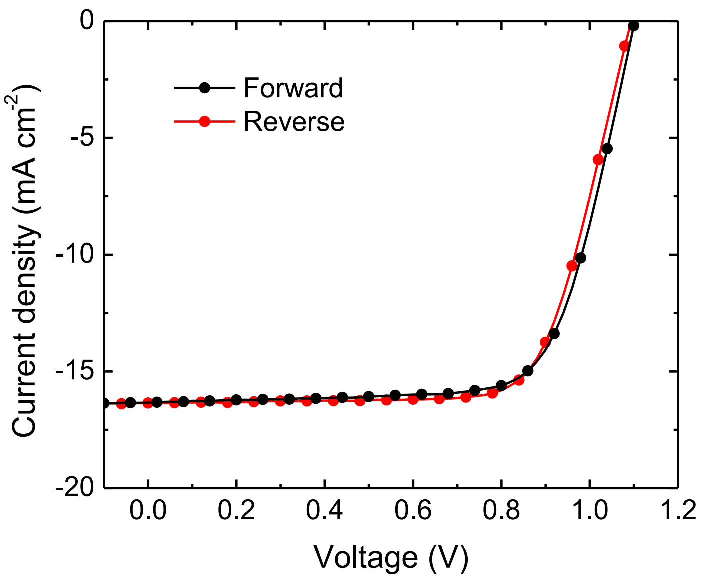

Supplementary Figure 9. *J-V* characteristics of the devices at different scan directions with the structure of glass/ITO/BrB-PEDOT:PSS/Q-2D/PCBM/BCP/Ag.

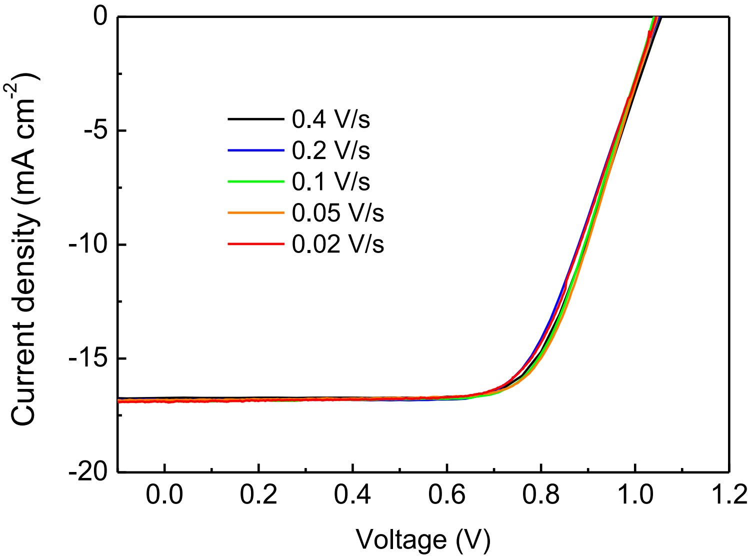

Supplementary Figure 10. *J-V* characteristics of the devices at different scan rates with the structure of glass/ITO/BrB-PEDOT:PSS/Q-2D/PCBM/BCP/Ag.

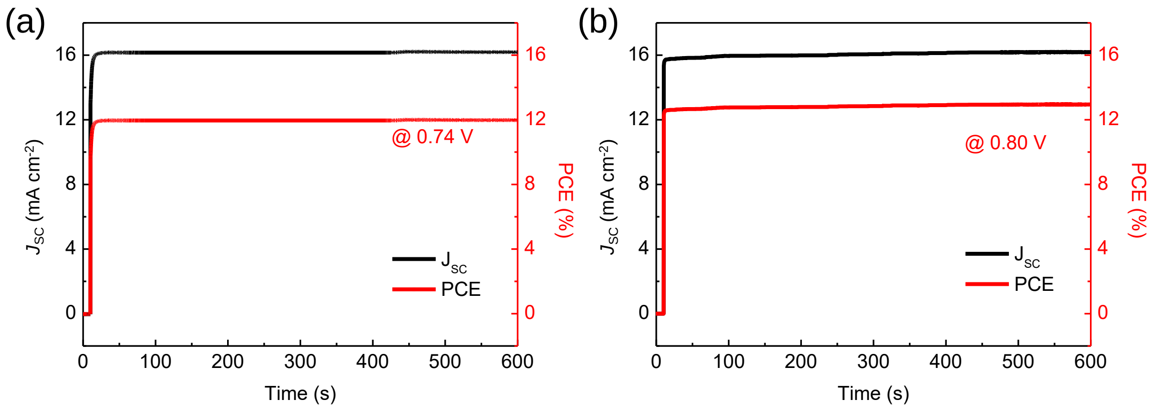

Supplementary Figure 11. Steady power out at maximum power point of the cells: (a) with PEDOT:PSS HTL; (b) with BrB-PEDOT:PSS HTL.

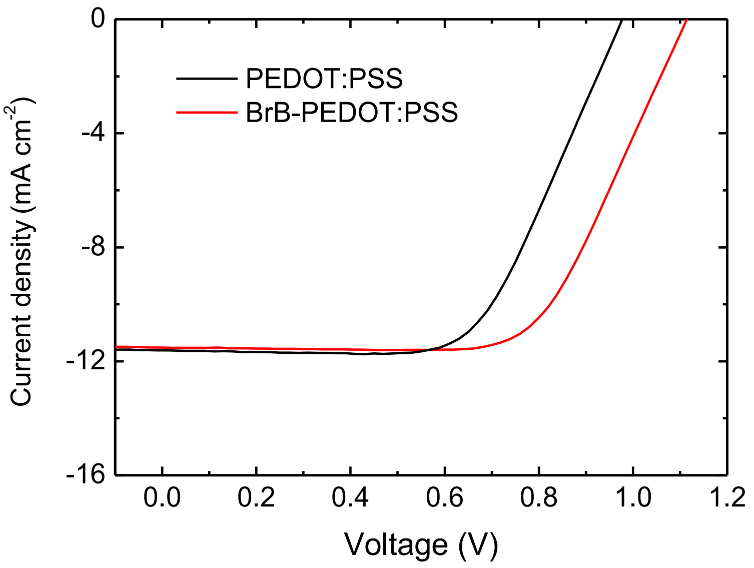

Supplementary Figure 12. The *J-V* characteristics of the devices with the structure of glass/ITO/HTL/Q-2D/PCBM/BCP/Ag. The HTL is PEDOT:PSS or BrB-PEDOT:PSS, the Q-2D perovskite film is with 5% Cs doping and the solvent is pure GBL for comparison.

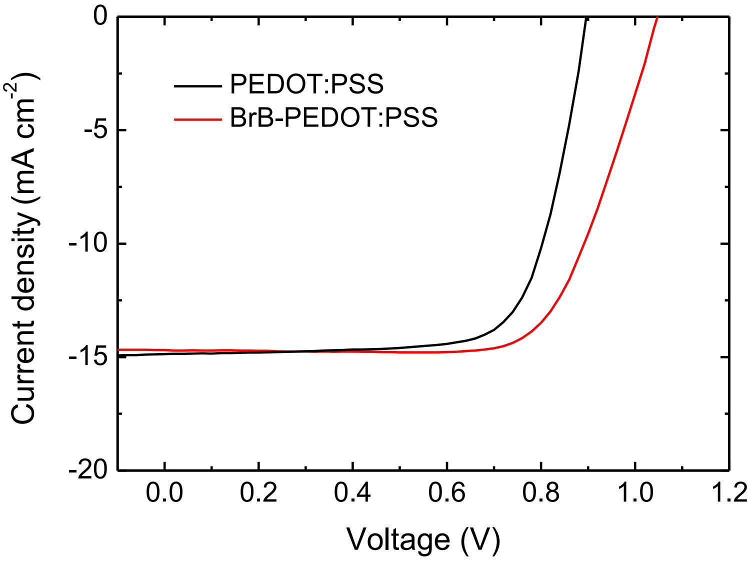

Supplementary Figure 13. *J-V* characteristics of the devices with the structure of glass/ITO/HTL/Q-2D/PCBM/BCP/Ag. The HTL is PEDOT:PSS or BrB-PEDOT:PSS, the Q-2D perovskite film is without 5% Cs doping, and the solvent is GBL:DMSO mixture (7:3, v/v).

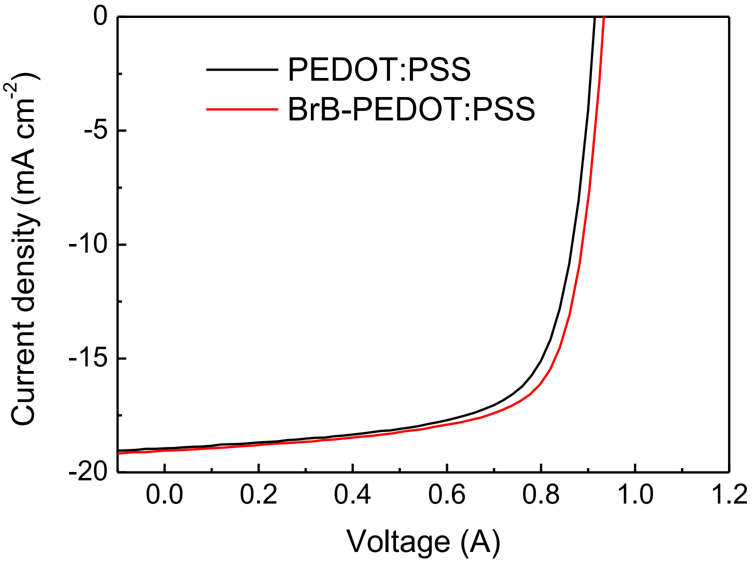

Supplementary Figure 14. *J-V* characteristics of the conventional 3D perovskite solar cells with PEDOT:PSS and BrB-PEDOT:PSS as HTLs. The device structure is glass/ITO/HTL/$MAPbI_3$/PCBM/BCP/Ag (where the HTL is PEDOT:PSS or BrB-PEDOT:PSS).

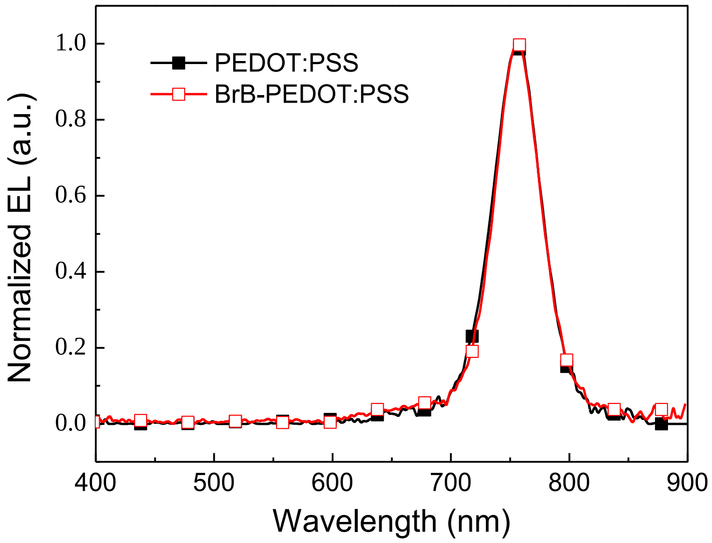

Supplementary Figure 15. EL spectra of devices with PEDOT:PSS and BrB-PEDOT:PSS as the HTL.

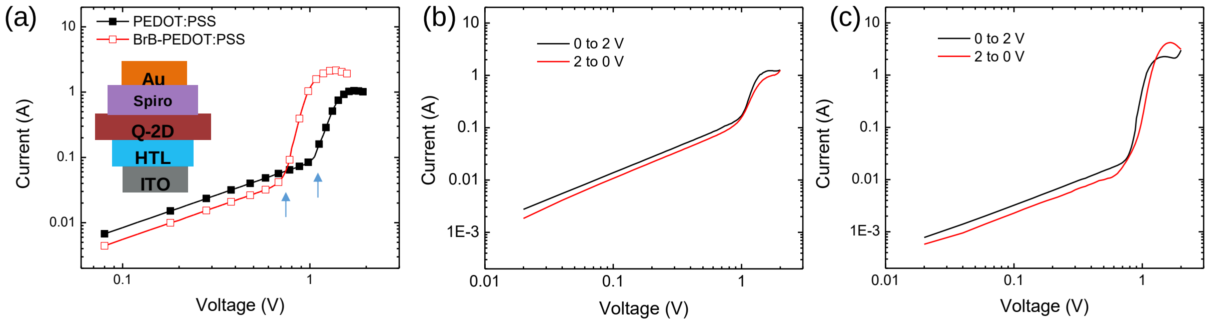

Supplementary Figure 16. (a) *J-V* characteristics of hole-only devices with PEDOT:PSS and BrB-PEDOT:PSS as the bottom HTL, respectively; The inset is the structure of the hole-only devices; *J-V* characteristics scanned in both reverse and forward directions to show the hysteresis: (b) device with PEDOT:PSS as the bottom HTL; (c) BrB-PEDOT:PSS as the bottom HTL.

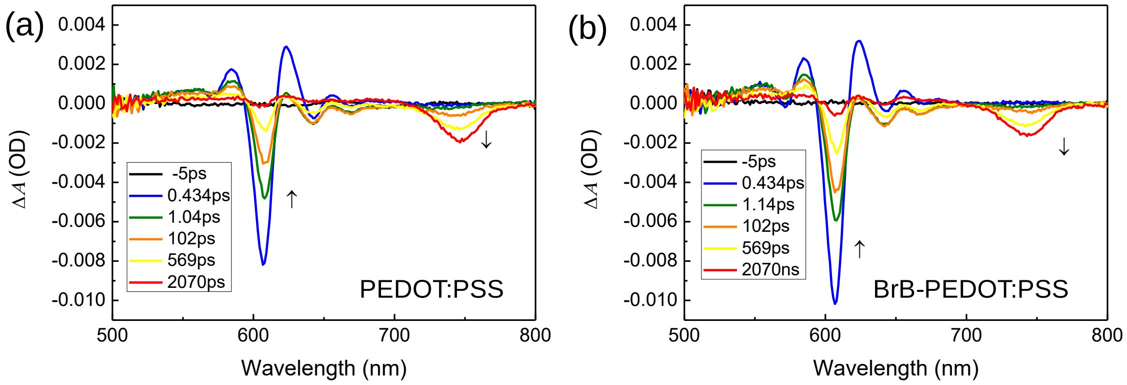

Supplementary Figure 17. Transient absorption spectra at different delay time of Q-2D perovskite films (400 nm thickness) deposited on: (a) PEDOT:PSS and (b) BrB-PEDOT:PSS under back-excitation at 480 nm.

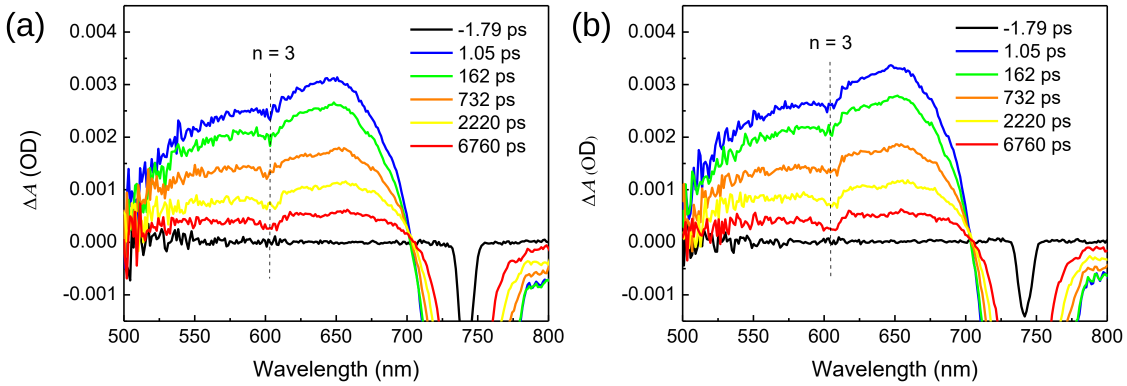

Supplementary Figure 18. Transient absorption spectra at different delay time of the Q-2D perovskite film (~ 400 nm thickness) deposited on (a) PEDOT:PSS and (b) BrB-PEDOT:PSS under front-excitation at 740 nm. The bleach peak at 605 nm is assigned to the n = 3 perovskite phase.

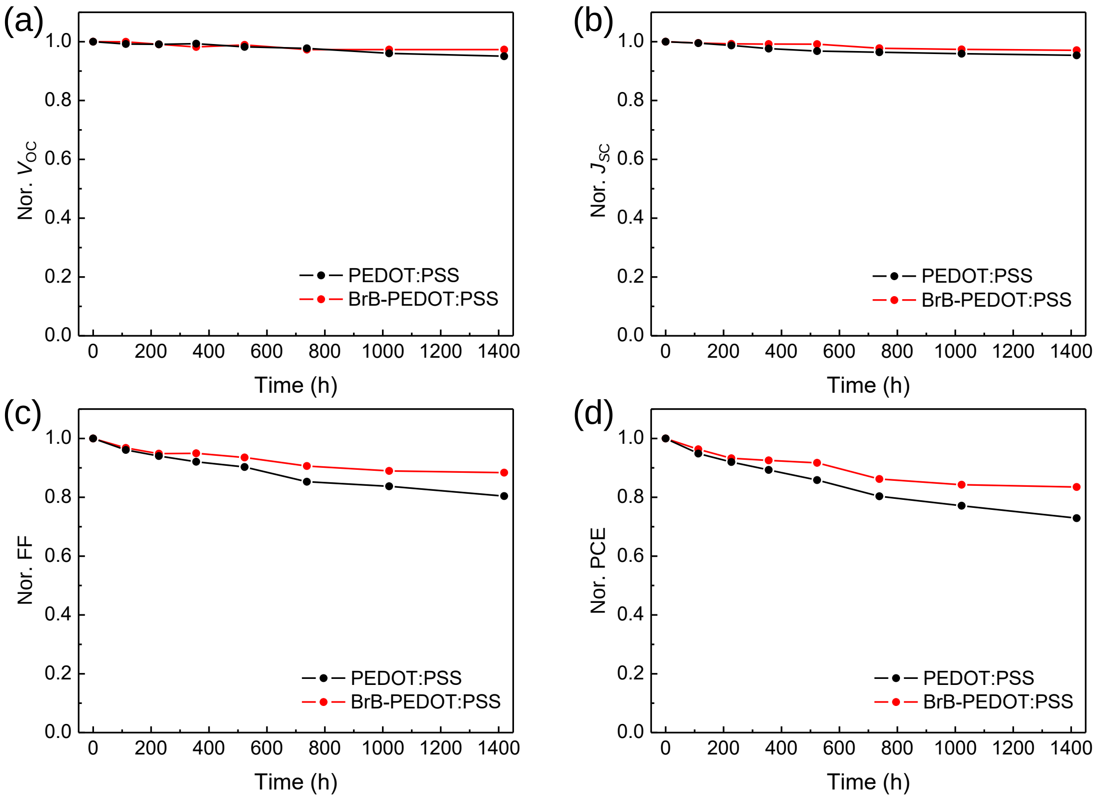

Supplementary Figure 19. Normalized (a) V_{OC}, (b) J_{SC}, (c) FF and (d) PCE of perovskite solar cells based on different HTLs as a function of storage time in dark in air without any encapsulation (25 °C, 20 % relative humidity).

Supplementary Table 1. Summary of solar cell structures of BA-based Q-2D perovskite (n < 10) and their photovoltaic performance reported in the literature.

Perovskite	Device configuration	V_{OC} (V)	J_{SC} (mA cm^{-2})	FF	PCE (%)
$(BA)_2(MA)_3Pb_4I_{13}$	FTO/PEDOT:PSS/Q-2D/PCBM/Al	1.01	16.76	0.74	12.51[2]
$(BA)_2(MA)_3Pb_4I_{13}$	ITO/PEDOT:PSS/ Q-2D/PCBM/BCP/Al	0.954	16.13	0.70	10.70[3]
$(BA)_2(MA_{0.8}FA_{0.2})_3Pb_4I_{13}$	ITO/PEDOT:PSS/Q-2D/PCBM/BCP/Al	0.999	18.12	0.71	12.81[3]
$(BA)_2(MA)_4Pb_5I_{16}$	ITO/PEDOT:PSS/Q-2D/PCBM/Al	0.9856	15.49	0.66	10.00[4]
$(BA)_2(MA)_3Pb_4I_{13}$	FTO/C_{60}/Q-2D/Spiro-OMeTAD/Au	1.06	11.70	0.43	5.38[5]
$(BA)_2(MA_{0.95}Cs_{0.05})_3Pb_4I_{13}$	FTO/TiO_2/Q-2D/Spiro-OMeTAD/Au	1.08	19.95	0.63	13.68[6]
$(BA)_2(MA)_2Pb_3I_{10}$	FTO/TiO_2/Q-2D/Spiro-OMeTAD/Au	0.93	9.42	0.46	4.02[1]
$(BA)_2(MA)_2Pb_3I_{10}$	ITO/PEDOT:PSS/Q-2D/PCBM/BCP/Al	0.97	12.79	0.55	6.89[7]
$(BA)_2(MA)_3Pb_4I_{13}$	ITO/PEDOT:PSS/Q-2D/PCBM/BCP/Ag	0.98	14.71	0.61	8.79[7]
$(BA)_2(MA)_3Pb_4I_{13}$	ITO/PEDOT: PSS/Q-2D/C_{60}/BCP/Ag	1.01	15.8	0.69	11.0[8]
$(BA)_2CsPb_2I_7$	FTO/c-TiO2/Q-2D/spiro-OMeTAD/Au	0.95	8.88	0.57	4.84[9]
$(BA)_2(FA)_2Pb_3I_{10}$	ITO/PEDOT:PSS/Q-2D/PCBM/BCP/Ag	0.98	11.89	0.59	6.88[10]
$(BA)_2(MA_{0.95}Cs_{0.05})_3Pb_4I_{13}$	ITO/BrB-PEDOT:PSS/Q-2D/PCBM/BCP/Ag	1.11	17.08	0.73	13.74 (This work)

Supplementary Table 2. Fitting parameters for *J-V* characteristics of the devices in the dark (glass/ITO/HTL/$MAPbI_3$/PCBM/BCP/Ag (where the HTL is PEDOT:PSS or BrB-PEDOT:PSS) with one-diode equivalent circuit model.

HTL	J_{sat} (mA/cm^2)	n_{if}
PEDOT:PSS	1.41×10^{-7}	2.22
BrB-PEDOT:PSS	1.33×10^{-9}	1.89

Supplementary Table 3. Fitting parameters for the kinetics probed at 740 nm shown in Figure 5c. The kinetics are fit by a multiple-exponential function, $\Delta A(t) = A_1 \exp(-t/\tau_1) - C_1 \exp(-t/\tau_{et})$, where A_1 and C_1 are the amplitudes; τ_1 is the decay time constant and τ_{et} is the electron transfer time constant.

HTL	τ_{et} (ps)	τ_1 (ps)
PEDOT:PSS	742	20990
BrB-PEDOT:PSS	676	12354

Supplementary Table 4. Fitting parameters for the kinetics probed at 605 nm shown in Figure 5d. The kinetics are fit by a multiple-exponential function, $\Delta A(t) = A_1 \exp(-t/\tau_1) - C_1 \exp(-t/\tau_{ht})$, where A_1 and C_1 are the amplitudes; τ_1 is the decay time constant and τ_{ht} is the hole transfer time constant.

HTL	τ_{ht} (ps)	τ_1 (ps)
PEDOT:PSS	505	26074
BrB-PEDOT:PSS	457	8987

Supplementary References:

(1) Cao, D. H., Stoumpos, C. C., Farha, O. K., Hupp, J. T. & Kanatzidis, M. G. 2D Homologous Perovskites as Light-Absorbing Materials for Solar Cell Applications. *J. Am. Chem. Soc.* **137**, 7843-7850 (2015).

(2) Tsai, H. H. et al. High-efficiency two-dimensional Ruddlesden-Popper perovskite solar cells. *Nature* **536**, 312-317 (2016).

(3) Zhou, N. et al. Exploration of Crystallization Kinetics in Quasi Two-Dimensional Perovskite and High Performance Solar Cells. *J. Am. Chem. Soc.* **140**, 459-465 (2018).

(4) Soe, C. M. M. et al. Understanding Film Formation Morphology and Orientation in High Member 2D Ruddlesden-Popper Perovskites for High-Efficiency Solar Cells. *Adv. Energy Mater.* **8**, 1700979 (2018).

(5) Chen, Y. N. et al. Tailoring Organic Cation of 2D Air-Stable Organometal Halide Perovskites for Highly Efficient Planar Solar Cells. *Adv. Energy Mater.* **7**, 1700162 (2017).

(6) Zhang, X. et al. Stable high efficiency two-dimensional perovskite solar cells via cesium doping. *Energy Environ. Sci.* **10**, 2095-2102 (2017).

(7) Zhang, X. et al. Vertically Oriented 2D Layered Perovskite Solar Cells with Enhanced Efficiency and Good Stability. *Small* **13**, 1700611 (2017).

(8) Ma, C., Shen, D., Ng, T. W., Lo, M. F. & Lee, C. S. 2D Perovskites with Short Interlayer Distance for High-Performance Solar Cell Application. *Adv. Mater.* **30**, e1800710 (2018).

(9) Liao, J. F., Rao, H. S., Chen, B. X., Kuang, D. B. & Su, C. Y. Dimension engineering on cesium lead iodide for efficient and stable perovskite solar cells. *J. Mater. Chem. A* **5**, 2066-2072 (2017).

(10) Yan, J. et al. Highly oriented two-dimensional formamidinium lead iodide perovskites with a small bandgap of 1.51 eV. *Mater. Chem. Front.* **2**, 121-128 (2018).
